# Core Competencies for Pain Management: Results of an Interprofessional Consensus Summit

**DOI:** 10.1111/pme.12107

**Published:** 2013-04-11

**Authors:** Scott M Fishman, Heather M Young, Ellyn Lucas Arwood, Roger Chou, Keela Herr, Beth B Murinson, Judy Watt-Watson, Daniel B Carr, Debra B Gordon, Bonnie J Stevens, Debra Bakerjian, Jane C Ballantyne, Molly Courtenay, Maja Djukic, Ian J Koebner, Jennifer M Mongoven, Judith A Paice, Ravi Prasad, Naileshni Singh, Kathleen A Sluka, Barbara St Marie, Scott A Strassels

**Affiliations:** * Department of Anesthesiology and Pain Medicine, School of Medicine, University of California, DavisSacramento, California; † Betty Irene Moore School of Nursing, University of California, DavisSacramento, California; ‡ School of Education, University of PortlandPortland, Oregon; § Department of Medicine, Medical Informatics and Clinical Epidemiology, Oregon Health & Science UniversityPortland, Oregon; ¶ College of Nursing, University of IowaIowa City, Iowa; ** Department of Physical Therapy and Rehabilitation Science, University of IowaIowa City, Iowa; †† Department of Neurology, Johns Hopkins UniversityBaltimore, Maryland; ‡‡ Program on Pain Research, Education and Policy, Department of Public Health and Community Medicine, Tufts University School of MedicineBoston, Massachusetts; §§ Department of Anesthesiology & Pain Medicine, University of WashingtonSeattle, Washington; ¶¶ College of Nursing, New York UniversityNew York, New York; *** Department of Medicine—Hematology/Oncology, Feinberg School of Medicine, Northwestern UniversityChicago, Illinois; ††† Division of Pain Medicine, Stanford University School of MedicineRedwood City, California; ‡‡‡ Fairview Health ServicesMinneapolis, Minnesota; §§§ Health Pharmacy and Pharmacy Practice Division, University of Texas at AustinAustin, Texas; ¶¶¶ Lawrence S. Bloomberg Faculty of Nursing, University of TorontoToronto, Ontario, Canada; **** School of Health and Social Care, Faculty of Health & Medical Sciences, University of SurreySurrey, UK

**Keywords:** Pain, Pain Management, Clinical Competence, Competencies, Interprofessional, Curriculum, Education, Health Professions

## Abstract

**Objective:**

The objective of this project was to develop core competencies in pain assessment and management for prelicensure health professional education. Such core pain competencies common to all prelicensure health professionals have not been previously reported.

**Methods:**

An interprofessional executive committee led a consensus-building process to develop the core competencies. An in-depth literature review was conducted followed by engagement of an interprofessional Competency Advisory Committee to critique competencies through an iterative process. A 2-day summit was held so that consensus could be reached.

**Results:**

The consensus-derived competencies were categorized within four domains: multidimensional nature of pain, pain assessment and measurement, management of pain, and context of pain management. These domains address the fundamental concepts and complexity of pain; how pain is observed and assessed; collaborative approaches to treatment options; and application of competencies across the life span in the context of various settings, populations, and care team models. A set of values and guiding principles are embedded within each domain.

**Conclusions:**

These competencies can serve as a foundation for developing, defining, and revising curricula and as a resource for the creation of learning activities across health professions designed to advance care that effectively responds to pain.

## Introduction

Pain is the most common reason individuals visit a health care professional. Worldwide, inadequately managed pain is the source of major human and economic costs for patients, their families, and society 1. According to the Institute of Medicine (IOM), approximately 100 million Americans suffer from chronic pain at an estimated annual cost of approximately 600 billion dollars 2. This figure exceeds the cost of each of the nation's priority health conditions 3. These estimates do not include the considerable burdens of acute pain and cancer-related pain 1. Despite its importance in clinical practice, pain management receives little emphasis in the curricula of most prelicensure health care professional education programs 4–13. In its 2011 monograph on pain in America, the IOM noted, *“Curricula for all health professions are full, and advocates of many important causes compete for a greater share of students' and clinicians' valuable educational time. Yet despite the large role that care of patients with pain will play in their daily practice, many health professionals, especially physicians, appear underprepared for and uncomfortable with carrying out this aspect of their work. These professionals need and deserve greater knowledge and skills so they can contribute to the necessary cultural transformation in the perception and treatment of people with pain.”* 2

Historically, the value of core curricula for pain education was recognized in the 1990s by the International Association for the Study of Pain (IASP); Canadian health professions faculty used the IASP Core Curriculum to develop an integrated interprofessional pain curriculum and applied this to prelicensure education for health care professionals in that nation 14. As a result, Canadian students have demonstrated improved outcomes in their knowledge and beliefs about pain. Furthermore, the faculty believed that the curricula addressed several learning needs, such as improved integration of knowledge with clinical decision-making 15. The available IASP curricula were uniprofessional until 2012 when its prior curricula for each profession were updated and a separate interprofessional curriculum was developed 16. These advances in pain-related curricula took place concurrent with innovations in the educational process and evolving paradigms for teaching and learning. Such advances and innovations have reduced the prior emphasis upon factual knowledge that learners are expected to acquire, and increased the emphasis placed upon students' capacity to act effectively in complex, diverse, and variable situations 17.

The desired outcomes of the educational process, competency-based education (CBE), emphasizes the learner's capacity to successfully carry out tasks in the real world, rather than the capacity to absorb and recite content 18. CBE focuses on the desired performance characteristics of health care professionals 18, as opposed to what or how learners are taught. Thus, CBE shifts the metrics for judging the effectiveness of educational programs toward assessing the practical impact of education instead of simply its content or process 18.

The emphasis of CBE on outcomes of pain education echoes the rise of outcomes assessment more broadly in health care 19. The call for outcomes assessment as an integral part of person-centered pain care followed widespread recognition that the quality of care is not improved simply by accumulating and disseminating the best available evidence 20–22. Instead, excellence in person-centered care requires that clinicians respond to patients' needs and preferences in a compassionate, knowledgeable, and coordinated fashion 23. Assessment of competencies is more closely aligned with the reality (i.e., quality) of clinical care than is assessment of knowledge alone. Thus, both in the education of entry-level health care professionals, as well as in the modification of postgraduate clinician behavior, a shift toward assessment of quality and outcomes of care has given rise to a need to associate curricular content with competencies.

Inadequate education of health care professionals is a major and persistent barrier to safe and effective pain management. Despite the health professions' development of competencies in pain management for advanced learners 24, special populations 25, and specific types of pain 26, as well as the myriad guidelines and position articles on pain management issued by numerous professional bodies representing thousands of clinicians 27, core pain management competencies for prelicensure entry-level health professional learners have not yet been established. The absence of core competencies may in part be a reason for the paucity of pain education found in undergraduate programs. The limited pain education that is currently provided may be ineffective because it focuses on traditional impersonal topics such as anatomy and physiology that may have little direct relevance to the complex daily problems faced by patients, families, and clinicians 28.

A noteworthy and relevant trend in health care education is the recognition that increased collaboration and teamwork are necessary to improve the quality and safety of health care 29. The IOM, World Health Organization, and numerous professional groups envisage interprofessional education as an important part of preparing a workforce to practice collaboratively at a time when the number of patients with complex, long-term medical problems is expanding at an unprecedented rate 29. The shift from multidisciplinary/multiprofessional to interprofessional team pain care resonates perfectly with the present emphasis upon interprofessional education and practice.

To help bridge the gap between the compelling needs of persons in pain and the skills, knowledge, and values of the interprofessional health care team, a group of educators and clinicians was convened to undertake a structured interprofessional consensus process in order to develop core pain management competencies appropriate for prelicensure health care providers. Annex 1 includes a list of professions represented at the summit. “Prelicensure education” refers to the training period prior to obtaining initial licensure to practice in the chosen profession. Prelicensure education was chosen because it represents the foundational period of entry-level health professional education; however, application of these competencies may be relevant to clinical learners well beyond prelicensure training (e.g., post-licensure training or continuing education). Within an interprofessional team delivering person-centered care, each profession will carry out roles that require both common knowledge and specific educational content to support achieving competencies in a manner consistent with each profession's scope, emphasis, and role in health care.

## Methods

The structured process for identifying pain management core competencies for prelicensure learners took place in two phases from October 2011 through November 2012. During Phase I (October–July), an executive committee (EC) comprised of seven experts in pain management and education synthesized current evidence and existing profession-based competencies to develop a draft set of candidate competencies. During Phase II (August–November), an international competency advisory committee (CAC) of 22 members representing 10 professions reviewed all draft materials and then met in-person to finalize the draft materials and recommend a final set of consensus-based competencies.

### Phase I

The EC included seven leaders from multiple professions who collectively brought expertise in pain management, education science, and development of evidence-based consensus. This group collaborated over 10 months to identify literature and other relevant material for review, define key terms, and identify individuals to participate in the interprofessional summit as part of a CAC. The EC defined the initial structure and draft content of the competencies, as well as the topics and goals of the summit.

During Phase I, the EC examined the current state of existing core competencies and pain management education for health professional learners. Recommendations of key publications, existing curricula, and core competencies were solicited from members of the EC as well as from other key content experts. Foundational references identified from these sources served as a starting point for a literature review.

Electronic journal databases were searched using Pubmed and CINAHL. A partial list of search terms and other researched sources is included as Annex 2. The search strategy used pain-related terms (e.g., pain, pain management) in combination with education-related terms such as competency/competency-based education and curriculum. Health profession terms (e.g., medicine, nursing) and concepts such as consensus building and interprofessional education were also explored. The search was limited to English language articles. Emphasis was placed on identifying content focused on prelicensure learners, but not limited to that group. Moreover, an internet search using the Google search engine identified grey literature that was produced by government, academia, business, and industry sources to identify existing competencies, curricula, educational programs, and clinical guidelines on pain management. Grey literature refers to written material including reports that are difficult to access via conventional channels such as published journals but is considered an important source of information because it tends to be original and recent 30. This search identified multiple existing pain management curricula, as well as established competencies in related fields. A document produced by a group assembled by the American Geriatrics Society, “Multidisciplinary Competencies in the Care of Older Adults at the Completion of the Entry-Level Health Professional Degree” was employed as a template through the development process 31. Finally, a search was conducted of the following professional associations' Websites: American Academy of Pain Medicine, American Chronic Pain Association, American Pain Society, American Society for Pain Management Nursing, and IASP.

A comprehensive list of source material was created that included peer-reviewed and grey literature of existing curricula, competencies, and clinical guidelines. The EC identified and analyzed themes and key content of each source. Themes were grouped into the following basic categories as a starting point: What is pain? What is the context of pain? What does pain look like? What affects pain? How is pain relieved? The EC identified a natural and synergistic link with the topic areas addressed in the 2012 revised curricula prepared by the IASP 16 and mapped the domains to those topic areas (i.e., multidimensional nature of pain, pain assessment and measurement, management of pain, clinical conditions). The EC went on to review several iterations of the competencies and develop a list of 40 draft competencies under the four domains for the CAC to review and critique during the consensus summit.

### Phase II

The 29-person Expert Interprofessional Pain Competencies Consensus Group assembled for the summit included both the EC and the CAC members. The group members brought experience in clinical pain management; research and education in pain management; education science; curriculum development; interprofessional education and teamwork; and knowledge uptake. All group members were from the United States and Canada. A particular effort was made to include individuals who were active in pain management professional associations and other stakeholder groups. A full list of members is included in Annex 3. Veterinary medicine was included because of evidence from Canada suggesting that veterinary medicine students receive more extensive and effective prelicensure education on pain than other prelicensure health professionals and because of the extensive experience of these clinicians in treating nonverbal patients 5.

The EC provided the CAC members with a synthesis of the literature on competencies in pain management across the health professions for review. The group then convened for a 2-day *Summit for Interprofessional Consensus on Pain Management Competencies* in Sacramento, California, in August 2012. Each participant disclosed potential conflicts of interest and agreed to contribute feedback that was independent and objective. Members of the EC led the summit. The group reviewed the central concepts of CBE and agreed upon key terms to establish a common understanding of nomenclature before embarking on a critique of the draft documents and subsequent consensus building. The initial compilation of 40 draft competencies under the four domains were reviewed as a full group before beginning a series of small group discussions led by EC members utilizing the WorldCafé™ 32 model. This method offered each participant the opportunity to evaluate and respond to each of the initial competencies through a focused 20-minute dialogue with four to five other CAC members. The EC randomly assigned CAC members to four groups. Every group rotated through each of the four rooms, thereby allowing every CAC member to comment on all four domains and its associated competencies.

Following the WorldCafe™ sessions, the full summit group reassembled to review and discuss each domain including all the comments and findings collected from each group. A list of key terms and a set of core values that are embedded throughout the domains were also identified on Day 1. After the full group discussion, the EC met to synthesize and refine the competencies and presented a revised list to the full group at the beginning of the second day of the summit. An open voting process was used to confirm consensus among the full group on the content and structure of the domains and competencies.

Following the summit, EC members incorporated CBE terminology, reduced redundancies, and clarified language. The updated domains and competencies were sent to the CAC for review and refinement in October 2012. The final document reflects consensus on the review and endorsement of the core values, competencies, and definitions of key terms.

## Results

From the initial 40 draft competencies, 25 competencies applied to pain assessment and management were supported by the close of the summit. EC members further condensed the list to reduce redundancies within domains as well as to refine final competencies. The final list included 21 pain assessment and management core competencies under four domains. The competencies are outcome based and focus on actions health professional students should be able to perform in a variety of complex situations prior to completion of prelicensure training.

The first three domains address 1) the concepts and complexity of pain; 2) pain assessment; and 3) collaborative approaches to treatment. These domains highlight the foundational skills and knowledge each clinician should possess to identify, assess, and treat pain. The fourth domain focuses on the application of effective pain management in various populations and contexts. The full list of domains and competencies are listed in Box 1.

**Box 1 Pain management domains and core competencies**

Domain one

Multidimensional nature of pain: What is pain?

This domain focuses on the fundamental concepts of pain including the science, nomenclature, and experience of pain, and pain's impact on the individual and society.

1. Explain the complex, multidimensional, and individual-specific nature of pain.
2. Present theories and science for understanding pain.
3. Define terminology for describing pain and associated conditions.
4. Describe the impact of pain on society.
5. Explain how cultural, institutional, societal, and regulatory influences affect assessment and management of pain.

Domain two

Pain assessment and measurement: How is pain recognized?

This domain relates to how pain is assessed, quantified, and communicated, in addition to how the individual, the health system, and society affect these activities.

1. Use valid and reliable tools for measuring pain and associated symptoms to assess and reassess related outcomes as appropriate for the clinical context and population.
2. Describe patient, provider, and system factors that can facilitate or interfere with effective pain assessment and management.
3. Assess patient preferences and values to determine pain-related goals and priorities.
4. Demonstrate empathic and compassionate communication during pain assessment.

Domain three

Management of pain: How is pain relieved?

This domain focuses on collaborative approaches to decision-making, diversity of treatment options, the importance of patient agency, risk management, flexibility in care, and treatment based on appropriate understanding of the clinical condition.

1. Demonstrate the inclusion of patient and others, as appropriate, in the education and shared decision-making process for pain care.
2. Identify pain treatment options that can be accessed in a comprehensive pain management plan.
3. Explain how health promotion and self-management strategies are important to the management of pain.
4. Develop a pain treatment plan based on benefits and risks of available treatments.
5. Monitor effects of pain management approaches to adjust the plan of care as needed.
6. Differentiate physical dependence, substance use disorder, misuse, tolerance, addiction, and nonadherence.
7. Develop a treatment plan that takes into account the differences between acute pain, acute-on-chronic pain, chronic/persistent pain, and pain at the end of life.

Domain four

Clinical conditions: How does context influence pain management?

This domain focuses on the role of the clinician in the application of the competencies developed in domains 1–3 and in the context of varied patient populations, settings, and care teams.

1. Describe the unique pain assessment and management needs of special populations.
2. Explain how to assess and manage pain across settings and transitions of care.
3. Describe the role, scope of practice, and contribution of the different professions within a pain management care team.
4. Implement an individualized pain management plan that integrates the perspectives of patients, their social support systems, and health care providers in the context of available resources.
5. Describe the role of the clinician as an advocate in assisting patients to meet treatment goals.

During the course of discussions, core values that are integral to and embedded within each domain were identified. Participants felt that certain other principles, such as advocacy, collaboration, compassion, effective communication, and evidence-based practice, were relevant to all domains and competencies. Participants came to a consensus on the set of core values and principles that should be considered and incorporated into the development of pain management curricula and learning activities. The set of all core values/principles is presented in Box 2.

**Box 2 Core values/principles**

**Table:** 

•Advocacy	•Empathy
•Collaboration	•Ethical treatment
•Communication	•Evidence-based practice
•Compassion	•Health disparities reduction
•Comprehensive care	
•Cultural inclusiveness	•Interprofessional teamwork
	•Patient-centered care

The full summit group also stressed the importance of clearly defining key terms relevant to pain management and related competencies: pain, advocacy, comprehensive care, cultural inclusiveness, evidence-based practice, interprofessional teamwork, professional competencies, and social support system.

Several terms related to analgesia such as addiction, adherence, and misuse were also discussed at length. The group believed that these terms should be clearly defined as a resource for curriculum development. These terms, as well as many others, are provided on the project Website (http://www.ucdmc.ucdavis.edu/paineducation) and in the instructional resource material produced during the next phase of the project.

## Discussion

Through an interprofessional consensus process, core competencies in pain assessment and management (Box 1) were developed to address prelicensure pain management education in all major health care professions. These core competencies are consistent with the domain outline from the IASP pain curricula 16. Introducing pain education early in the preparation of health professionals emphasizes the value of improving quality of life and creates the potential to instill critical competencies that support the humanistic aspects of health care. Moreover, early education related to pain offers the opportunity to reverse the disparity between what students are taught and what they face in practice related to pain. Continuing to ignore pain as a substantial and critical part of the curriculum for health professionals stands in stark contrast to the importance of pain in society; that pain is the most common reason a person seeks clinical care; that undertreated, over-treated, or ineffectively treated pain greatly impacts major public health problems such as disability, prescription drug abuse, or the overall cost of health care; and that the cost of pain in terms of suffering is vast but immeasurable.

Although no comprehensive survey regarding pain education curricula across health care professions has been conducted in the United States, available evidence indicates that pain management training is widely inadequate across all disciplines 2,4–6,8,10–12. A survey of undergraduate pain curricula for health care professionals in the United Kingdom found pain education content for undergraduate health care professionals to be nominal and fragmented, accounting for less than 1% of program hours for some disciplines 4. A survey of Canadian prelicensure pain curricula for dentistry, medicine, nursing, occupational therapy, pharmacy, and physiotherapy students found that respondents representing the majority (67.5%) of health science programs could not specify designated hours for pain course content or clinical conferences 5. Veterinary medicine curricula were also surveyed for comparison and had five times more pain content than did medicine 5. The minimal number of designated pain hours is not surprising as a recent examination of Canadian requirements for nine entry-to-practice health science professions found pain competencies for only nursing (N = 9) and dentistry (N = 2) 32.

Limited data exist to clearly gauge the state of pain education in each individual profession; however, a review of available findings suggests in no instances are the offerings robust. A recent survey of pain-related content in educational institutions worldwide found that in spite of many achievements, medical school education in acute pain management was inadequate 12. The First National Pain Medicine Summit, convened in November 2009 by the American Medical Association's Pain and Palliative Medicine Specialty Section Council, found that training was poor or “not leading to competency” at both the undergraduate and residency levels 33. A recent study of 117 medical schools in the United States and Canada found that only four U.S. schools offered a required course on pain 6. A survey of 111 attending physicians, residents, nurse practitioners, and physician assistants working in community clinics all reported their pain management training as less than adequate 7. In a study of faculty within 16 U.S. Midwestern schools of nursing, approximately three fourths (72.9%) of the participants recalled being taught about pain management; but a minority (36.5%) believed that they were adequately prepared on the topic 9. In a faculty survey of accredited physical therapy education programs in North America, the most frequently reported amount of time spent on pain in the curriculum was 4 hours 8. The authors of a study designed to describe how and in what depth pain management is covered in U.S. pharmacy school curricula concluded that the topic of pain management is inadequately developed and poorly presented in many schools 10. Pain management, particularly for acute and postoperative pain, remains a core curriculum component in dentistry; yet, predoctoral and continuing education programs in chronic orofacial pain are limited 2. Despite efforts to address the need for professional education in pain management for psychologists 34, psychology training programs have been slow to adopt competency-based training in pain management 2. Data on the pain management training received by practitioners of complementary and alternative medicine are limited, but there are substantial variations found in pain education among chiropractors and acupuncturists 11.

This interprofessional consensus process was an inclusive endeavor, capitalizing on expertise from professionals with widely diverse backgrounds related to pain management. Through iterative review, including discussions and identification of central issues of agreement and disagreement, this systematic and collaborative process achieved a cohesive outcome. The process complemented efforts of the IASP to revise uniprofessional curricula and develop interprofessional curricular content. The active participation of members of the IASP Education Initiatives Working Group and the Chair of the subgroup that developed IASP's Interprofessional Pain Curriculum Outline [J. W.-W.] supported communication between, and alignment of, the two undertakings. Consequently, the competencies produced by this project parallel the structure of the interprofessional and uniprofessional pain curricula developed by IASP for various professions 16.

Our process and the resultant competencies have limitations. Although the panel members were chosen to achieve diversity and broad expertise, its composition may neither adequately represent the full spectrum of professions involved in pain management nor all views held by individuals within a single profession. The competencies are inclusive of a wide range of populations, settings, and conditions; however, additional competencies are necessary for other subpopulations such as children, older persons, or individuals with special cultural considerations that were not addressed in this initial effort. Moreover, the evidence review was limited to English language publications. While the competencies could serve as a global resource, their origin from a strongly North American perspective leaves open the need for adaptation to other languages, cultures, and value systems, as well as consideration of other national and regional concerns. In the absence of scientific evidence or endorsed professional standards, the expert panel rendered opinion that was ratified by consensus. As such, these competencies may help to bridge the gap between knowledge, learning, and clinical performance.

The panel faced a number of challenges. An early challenge was in defining the terms “interprofessional” (applying to health professions learning together) and “competency” (focusing on measurable outcomes of learning). In order to define competencies that were applicable across clinical professions involved in pain management, assembling a panel from diverse professions and disciplines was critical, but represented a challenge given the distinct perspectives and areas of clinical expertise of the participants. The in-person meeting with small and large group discussions, and use of a consensus facilitator helped participants work with various perspectives and aided identifying areas of consensus. Defining competencies related to the areas of evaluation and management was also challenging because learners in differing stages of training may have little or no direct clinical care, and different professions vary in their exposure of students to individuals with pain and their support systems. Our group therefore focused on core concepts necessary to effectively address pain and left implementation, including methods of teaching and evaluation, to users of the competencies. We also found it challenging to define the competencies in ways that would be measurable. Inclusion of experts in education and curriculum development was critical for reframing a number of the competencies in ways that facilitated measurement. Finally, the initial list of competencies generated by the group was quite lengthy. An iterative, consensus-building process facilitated defining a more concise “core” set of competencies. Nonetheless, it is our hope that these competencies will undergo further rigorous examination and refinement.

Although the need for basic expected competencies in pain management seems obvious, to our knowledge, these core competencies represent the first of their kind. Much work is needed to integrate the competencies into the education of health professionals and to evaluate impact. The domains and competencies are offered as a model and starting point for prelicensure pain management education in all health care professions. Developing these core competencies required focus on a specific period of clinical education and the prelicensure period was felt to be an important starting point; however, these competencies may be applicable to post-licensure education as well. They are neither exhaustive nor tailored to the specific needs of any one profession. Instead, they represent a minimum standard that may be variably emphasized in each educational undertaking, depending on each profession's unique needs, roles, and expectations as well as those for each educational program and institution.

We urge licensure, accreditation, certification, education, and policy governing bodies to engage in this important process and to consider these competencies when establishing standards. Curriculum developers across the health sciences are encouraged to evaluate their current educational content and adopt and test these competencies. It is envisioned that the competencies will be incorporated into learning activities that will be implemented through a myriad of didactic and case-based learning opportunities woven throughout the formative stages of professional development for future health care students. Exactly how these core competencies will be incorporated into diverse curricula within and across all health professions is not clear and will likely differ between professions. Mapping these competencies with existing curricula may help identify gaps or areas for improvement. They also offer a means of analyzing whether or not health professional curricula cover critical content related to pain assessment and management, and help guide curricular outcomes in this area.

## Conclusions

These consensus-based core competencies for pain management provide a basis for improving the culture and context of care for adults and children with acute and chronic pain or pain at end of life. They apply to all major clinical professions involved with pain management, although they target prelicensure education they apply across the spectrum from prelicensure to continuing education.

## Annex 1 Professions represented at the pain management competencies summit

**Table:**

Profession
Acupuncture
Dentistry
Education science
Medicine
Nursing
Pharmacy
Physical therapy
Psychology
Social work
Veterinary medicine

## Annex 2 Literature review terms and other resources

**Table:**

Electronic databases search terms
Clinical competence
Acupuncture/education
Competency-based education
Curriculum
Education
Education, dental
Education, medical
Education, medical, undergraduate
Education, nursing, baccalaureate
Education, nursing, graduate
Education, pharmacy
Educational status
Knowledge
Models, educational
Pain
Pain management
Physical therapy specialty/education
Psychology/education
Social work/education
Students, dental
Students, medical
Students, nursing
Students, pharmacy
Grey literature search
Agency for Healthcare Research and Quality
Center for Nursing Excellence in Long-Term Care, Geriatric Pain
City of Hope Pain & Palliative Care Resource Center
Institute of Medicine
Interprofessional Education Collaborative
Johns Hopkins University, School of Medicine Pain Curriculum
Maryland Board of Nursing, Pain Management Nursing Role/Core Competency A Guide for Nurses
Pain & Policy Studies Group (PPSG) at the University of Wisconsin Carbone Cancer Center
Tufts University, Pain Research, Education, and Policy (PREP) program curriculum
University of Toronto Centre for the Study of Pain
US DHHS National Guidelines Clearinghouse
Virginia Commonwealth University, Pain Education Curriculum
Professional associations
American Academy of Pain Medicine
American Association of Colleges of Nursing
American Chronic Pain Association
American Pain Society
American Society for Pain Management Nursing
Association of American Medical Colleges
International Association for the Study of Pain

## Annex 3 Expert interprofessional pain competencies consensus group

**Table:**

Executive committee	
Ellyn Arwood, EdD, University of Portland	Beth Murinson, MD, PhD, Johns Hopkins University
Roger Chou, MD, Oregon Health & Science University	Judy Watt-Watson, RN, MSc, PhD, University of Toronto
Scott M. Fishman, MD, University of California, Davis*	Heather M. Young, PhD, RN, FAAN, University of California, Davis*
Keela Herr, PhD, RN, AGSF, FAAN, University of Iowa	
Competency advisory committee	
Debra Bakerjian, PhD, FNP, University of California, Davis	
Jane Ballantyne, MD, University of Washington	
Steven Graff-Radford, DDS, Cedars-Sinai Medical Center	
Daniel B. Carr, MD, FABPM, Tufts University	
Molly Courtenay, PhD, MSc, BSc, Cert. Ed, RN, University of Surrey	
Maja Djukic, PhD, RN, New York University	
Steve Given, DAOM, Lac, American College of Traditional Chinese Medicine	
Debra Gordon, RN, DNP, FAAN, University of Washington	
Robin Kennedy, PhD, California State University Sacramento	
Ian J. Koebner, MSc, MAOM, LAc, University of California, Davis	
Nancy E. Lane, MD, University of California, Davis	
Judith Paice, PhD, RN, Northwestern University	
Ravi Prasad, PhD, Stanford University	
Bruno Pypendop, DrMedVet, DrVetSci, Dipl. ACVA, University of California, Davis	
Joanna Rowe, PhD, RN, Linfield College	
Todd Semla, PharmD, Northwestern University	
Naileshni Singh, MD, University of California, Davis	
Kathleen A. Sluka, PhD, PT, University of Iowa	
Barbara St. Marie, PhDc, RN, ANP, GNP, ACHPN, Fairview Health Services, Minneapolis, MN	
Bonnie Stevens, RN, PhD, University of Toronto	
Scott Strassels, PharmD, PhD, The University of Texas at Austin	
Barton L. Wise, MD, MSc, FACP, University of California, Davis	

* Project directors.
